# Beyond diversity, equity, and inclusion: American Dental Education Association's role in inclusivity, humanism, and leadership

**DOI:** 10.1002/jdd.13732

**Published:** 2025-05-27

**Authors:** Herminio L. Perez, Ana N. López Fuentes, Wendy Scripps

**Affiliations:** ^1^ Department of Restorative Dentistry, Rutgers School of Dental Medicine Newark New Jersey USA; ^2^ School of Dental Medicine University of Puerto Rico San Juan Puerto Rico USA; ^3^ Department of Ecological Sciences/Community Dentistry, School of Dentistry University of Alabama at Birmingham Birmingham Alabama USA

**Keywords:** cultural diversity, dental education, minority groups, professional responsibility

## Abstract

In 2022, the American Dental Education Association (ADEA) launched the first‐ever dental education‐wide climate assessment survey to establish baseline data on diversity, equity, and inclusion (DEI). This article aims to highlight the historical role of ADEA in supporting oral health education while building the inclusive capacity of leaders and advancing its organizational mission and vision in promoting DEI. The survey is a significant step in assisting academic dentistry in promoting a more humanistic environment while measuring the perception of students, faculty, staff, and leadership regarding DEI.

ADEA has significantly contributed to advancing dental education through data collection and the development of initiatives that enhance DEI across dental schools and allied education programs in the United States and Canada. The ADEA's efforts underscore its commitment to enhancing diversity, equity, and inclusion, aligning with its broader mission to improve oral health education.

## 1 INTRODUCTION

The issue of underrepresentation in dentistry, particularly among marginalized communities, has been extensively documented. Dr. William J. Gies, widely recognized as the pioneer of modern dental education, published a seminal study in 1926 that highlighted this disparity. Titled *“Dental Education in the United States and Canada: A Report to the Carnegie Foundation for the Advancement of Teaching,”* Dr. Gies notably created awareness and acknowledged the lack of diversity within the dental profession and advocated for its enhancement. His insights remain pertinent as efforts persist to promote diversity and equity across all aspects of dental education and practice. In his work, he stated:

“Economics, social, and educational conditions long conspired to prevent Blacks from entering the profession of health service. The creation of departments or schools for training them in the health service, in state universities, or universities having adequate endowments, should appeal strongly to the citizens of states containing large Black populations.^1^ ^(p93)^”

Dr. William J. Gies played a pivotal role in the establishment of the American Association of Dental Schools (AADS), and the advancement of dental education. In 1922, he proposed the amalgamation of several prominent dental organizations—namely, the Canadian Dental Faculties Association, the American Institute of Dental Teachers, the National Association of Dental Faculties, and the Dental Faculties of American Universities. As a testament to his leadership and vision, Dr. Gies was appointed as the temporary president, guiding the organization through its formation until its completion in September 1923.^2^

Founded in 1923 as the AADS, the American Dental Education Association (ADEA) has evolved into the leading advocate for dental education. ADEA represents dental educators, oral health professionals, and students, serving as a pivotal voice in shaping the future of dental education. Central to ADEA's mission is its vision of cultivating a well‐prepared and diverse oral health workforce that enhances the health of individuals and communities nationwide.

In 2022, the ADEA launched the first‐ever dental education‐wide climate assessment survey with the purpose of collecting baseline data on diversity, equity, and inclusion (DEI). The data collected exposes the need to address critical themes that can impact the educational experience of all involved in it. Among the themes identified were the lack of inclusion and belonging on campus, deficit of faculty of color, microaggressions, differential treatment of students of color, and the need to enhance diverse students and faculty of color.^3^ The survey and its findings are a major step in assisting academic dentistry in promoting a more humanistic environment while measuring the perceptions of students, faculty, staff, and leadership regarding DEI.

Throughout the years, ADEA has provided solutions to members and institutions making a difference. Some examples are providing meaningful resources, as well as fostering and leading collaborations and partnerships. Furthermore, ADEA has made significant contributions to DEI in dental education by driving innovation and excellence. Recruiting and retaining inclusive high‐quality faculty and staff, and promoting a culture of inclusivity, diversity, and equity in oral health education are other examples of ADEA's contribution.^4^

ADEA's value proposition is one that is forward‐thinking and ambitious with the goal to create an impact in oral health education. Many pioneers have contributed to the institution's mission and value proposition. This article aims to highlight the historical role of the ADEA in providing and supporting oral health education while considering the organization's mission and vision. In addition, this article highlights the characteristics of an inclusive leader. ADEA has played an important role not only in advancing dental education in various aspects but also in collecting data and developing important initiatives that promote DEI at the United States and Canadian dental schools and allied dental education programs.

### 1.1 Driving innovation and promoting DEI in oral health education

ADEA has relied on the steadfast support of trailblazers who embraced the organization's mission and vision, dedicating their time, expertise, and passion. One such pioneer was Dr. Jeanne Sinkford, who in 1991 was appointed Special Assistant to the Executive Director of AADS. With a distinguished career as dean of Howard Dental School from 1971 to 1991, Dr. Sinkford became a pioneer of diversity programming and policies. These initiatives were crucial in helping dental schools adapt to the evolving US demographic landscape and tackle the issue of underrepresented minority student enrollment in dental programs.^5^

In her commitment to fostering a diverse dental community, Dr. Sinkford developed a significant array of resources that are invaluable to all members of the dental profession. For example, in 2011, Dr. Sinkford published *Growing our Own: The ADEA Minority Dental Faculty Development Program*.^6^ The publication presents a framework that emphasizes diversity as a core value essential to enhancing the academic environment's quality for all students. In this publication, the authors advocate for the integration of diversity across policies and programs, aiming to cultivate a more inclusive educational landscape.

To address gender equality and foster leadership in global health, Dr. Sinkford founded the International Women Leadership Conference (IWLC). Since its inaugural conference in France in 1998 and most recently in 2024, the IWLC has been a pivotal platform addressing critical gender issues. These include enhancing female participation in dental education, advancing women in academic and research roles, and bolstering international leadership and research capabilities among women worldwide.^5^

In supporting the recruitment and retention of a diverse faculty and staff, ADEA published the 2020, *ADEA Faculty Diversity Toolkit*.^7^ This publication came in response to the lack of faculty diversity in dental education. This comprehensive document serves as a guide for dental schools and allied dental programs, offering best practices to recruit and retain historically underrepresented and marginalized faculty members. It provides strategic insights and resources to support institutions in fostering a diverse faculty body.

In 2022, The Journal of Dental Education launched a Special Edition focusing on Diversity, Equity, Inclusion, and Belonging. The edition presents a compilation of twenty‐three articles addressing current challenges in dental education and proposing actionable strategies for fostering significant change. It provides a platform to drive conversations and initiatives aimed at advancing diversity, equity, inclusion, and a sense of belonging within the dental community.

In addition to these publications, the ADEA has actively supported and educated admission officers in implementing a Holistic Admission & Review process. This initiative aims to assist dental schools in evaluating candidates as multifaceted individuals, considering their diverse competencies and experiences.^8^

In 2022, the ADEA published “Slow to Change: HURE Groups in Dental Education,” a comprehensive review addressing Historically Underrepresented Racial and Ethnic (HURE) applicants and graduates in dental education. This publication serves as a vital resource for dental schools and supports ADEA's ongoing dialogue with numerous stakeholders on HURE issues.

Dr. Karen West, President and CEO of ADEA, issued a compelling call to action in the publication, reaffirming ADEA's dedication to advancing dental education. Her statement resonates: *“As the Voice of Dental Education, we are with you to help lead. Let our solutions be systemic and our approaches daring and innovative to ensure that dental education and dentistry are accessible to all persons*.^9^ ^(p9)^ *”*

ADEA's commitment to promoting diversity and equity within dental education and practice is evident through these resources, which provide essential tools and insights to advance inclusive practices across the profession. Since its inception, ADEA has prioritized DEI and belonging in dental education, continually striving to cultivate an environment that embraces individuals from all backgrounds. This ongoing dedication ensures equal opportunities for advancement and success within the dental profession.

### 1.2 On Gies Impact

Dr. William J. Gies played an important role in dental education. In its *“Dental Education in the United States and Canada: A Report to the Carnegie Foundation for the Advancement of Teaching,”* he devoted chapter number five to discussing the deficiency of dental service for the black population. The chapter highlights the number of graduating black dentists and their distribution in the community. The lack of black dentists and its impact on the black population and lack of services.^1^

Despite it being more than 95 years since the Gies report, the issue of underrepresentation persists. In 2022, ADEA hosted the Men of Color in the Health Professions Summit. The gathering unites a diverse array of thought leaders to exchange ideas and develop strategies to combat the ongoing underrepresentation of men of color not only in dental professions, but also in fields like medicine, pharmacy, and other health‐related careers.^10^

### 1.3 Beyond DEI: inclusivity and humanism

Over the recent years, DEI has risen to the forefront of organizational priority, realizing the importance of focusing efforts on it and the positive outcomes that have come with it. Studies have underscored the significance of social belonging in cultivating commitment and engagement, essential elements for driving meaningful impact within both organizational and professional realms. Despite substantial investments—reaching approximately eight billion dollars in the US businesses alone in 2019 – a concerning 40 percent of individuals still reported feelings of isolation. This highlights a critical gap in the practice of intentional inclusivity within workplaces.^11^

Each facet of DEIB—Diversity, Equity, Inclusion, and Belonging—plays an indispensable role in catalyzing transformative change within the communities we serve. Whether it's within the confines of our own organization, our professional networks, or the broader societal landscape, embracing DEIB principles is not just a moral imperative, but also a strategic necessity in fostering a truly inclusive environment.

To cultivate a healthcare workforce reflective of the communities we serve, it's imperative that we begin by acknowledging the rich tapestry of diversity within those communities. This initial recognition serves as the foundation for advancing toward the next crucial step: inclusion. In 2008, a study conducted pointed out that the lack of inclusion is the cause of the lack of diversity and vice versa.^12^

As previously mentioned, each component of DEIB is a vital thread in the fabric of the community we serve. Each element represents a unique aspect of an individual's experience that can impact their sense of inclusiveness and belonging. For that reason, each one of these components works together and not independently unless it is to evaluate and enhance its impact on the group members. This is the ultimate way to create Humanism in oral health education.

In 2017, the Journal of Dental Education highlighted a strategic framework to assess the humanistic culture of dental schools. An essential article in that edition is the study conducted at the University of Michigan by Murdoch‐Kinch et al which assesses the culture and climate for diversity and inclusion and the humanistic learning environment for students, faculty, and staff.^13^

In its guest editorial address, Dr. Nader Nadershahi made a call to action to oral health leaders and educators by saying that *“a significant part of our role as educators is to develop, measure and constantly refine our school's culture in order to meet the strategic priorities and goals”*.^14^ ^(p10)^

In 2024 in her guest editorial address, “Oral Health Education and the Next 100 Years”, Dr. Ana López Fuentes commented: “Together, we can cultivate an oral health landscape that is more inclusive, more culturally competent, and ultimately more compassionate in the care of the patients entrusted to us over the next 100 years. We must continue to focus on cultivating a psychologically safe and inclusive environment in academic dentistry and patient care. We must continue to employ structural competencies enhancing institutional efforts to cultivate a humanistic learning environment.^15^ ^(p267)^”

### 1.4 Build the inclusive leadership capacity to create and value diversity

For the past years, and because of the social unrest in the United States, major organizations, including dental organizations, have focused their intentions on highlighting the importance and value of diversity while building leadership capacity to understand how critical it is to our dental schools and the community that they serve.

Motivation is associated with a managerial and leadership skill that is considered the force that influences and directs behavior, and it comes primarily from within the individual. Since it is an essential leadership skill, leaders must provide an environment that promotes a balance between intrinsic motivation (personal, internal) and extrinsic motivation (job environment and outcomes).^16^

Leadership motivation and participation play a vital role in creating a balance between intrinsic and extrinsic motivation, with the latter supporting changes in behavior. If motivation involves the engagement of the leadership in programs or initiatives that promote and cultivate inclusiveness and diversity, then dental organizations need to get on board to build their leadership capacity to value diversity.

A way to build the capacity is by educating leadership about what diversity is, how it looks at their respective institutions, and how they can create a positive contribution to the profession of dentistry and society at large. However, contrary to what might seem obvious, training does not serve the same purpose as education. Various studies concluded that diversity training does not change attitudes, let alone behaviors of the individual.^17^ Instead, it is more effective to engage leaders in creating programs that positively impact diversity, fostering social accountability.

The engagement of leaders in fostering diversity and inclusivity has been an essential topic of discussion when it comes to changing behaviors and perceptions of leaders. A recent study shows that leaders can initiate changes in diversity and inclusion if they engage in two behaviors; first, they can do so by fostering an environment where group members can express their unique viewpoints and perspectives. Second, they can make change by cultivating the value of diversity.^18^ This practice is an alternative to training programs. Involving a shift in a leader's behavior can be the solution needed to cultivate the motivation and value of diversity, impacting their perceptions and beliefs. It is for that reason that ADEA has contributed with a vast array of resources to educate members of the oral health education to learn about the community within our institutions and its impact on the community we serve.

Since 1994, ADEA has supported and developed programs that built the capacity of future leaders. For example, the ADEA Leadership Institute was created to provide opportunities to promising mid‐career faculty and administrators to learn about personal and interpersonal competencies for leadership, legislative, and administrative competencies while being mentored and guided by established leaders in oral health education.^19^ ADEA's commitment to leadership extends to dental and allied dental students through programs like the Student Diversity Leadership Program. This initiative engages participants in identifying resources to enhance their leadership styles and achieve their goals.^20^

### 1.5 Building an inclusive leader

An individual's perception of situations they encounter is highly influenced by their beliefs and life experiences, which significantly impact the definition of diversity and how it permeates various aspects of leadership in a higher education institution. Furthermore, these individual experiences can inform how diversity will be enhanced in the institution, impacting multiple aspects of the profession, including students, faculty, healthcare workforce, and access to care.

*“People bring patterns of behavior to the workplace that have roots in early life*.^21^ *”* Therefore, it is critical to educate leaders and to make them aware of the value of diversity and its impact on the community they serve academically and clinically to build their own leadership capacity.

Leadership is a complicated term to describe. Despite being defined as a process where an individual influences others to achieve a common goal. Many researchers have tried to develop an actual definition that would encompass everything that comprises being a leader. Considering leadership as a combination of traits and processes helps to distinguish key talents and behaviors of a leader, Northouse identifies four types of leadership: transformational, authentic, servant, and adaptive.^22^

A leader who is aware of his attitudes and approaches to diversity is considered an inclusive leader. This type of leader is characterized by promoting inclusive experiences across multiple identities to create organizations, workgroups, and communities where diversity is an advantage to the community that they serve.^23^

A leader in healthcare with these traits of inclusiveness and taking into consideration the value of diversity will have the potential to develop a workgroup environment that makes an impact in the healthcare workforce including students and faculty while providing the foundations to foster a culture where access to care will be an outcome.

### 1.6 Recommendations

There are different ways in which we can impact the experience of our students and peers in our academic environments. We as faculty members are all role models and as such, we have a responsibility to foster inclusive environments by making intentional efforts to learn and work on making our institutional climates ones that embrace inclusivity and belongingness. ADEA has a wealth of resources and offers opportunities to all its members and it is our task to familiarize with all that information and utilize them effectively to enhance the academic environment and enrich the educational experience of all involved in oral health education.

## 2 CONCLUSION

Throughout the years, ADEA has played a critical role in not only gathering data to assess the current landscape of oral health education but also in educating leaders, faculty, and students about the importance of diversity, equity, inclusion, and belonging. ADEA has also been instrumental in building the leadership capacity of oral health educators to actively promote inclusivity within their academic institutions. Within the context of DEIB, ADEA has identified inspiring leaders who have become trailblazers. Each of those trailblazers exemplified characteristics that have profoundly influenced the organization's commitment to diversity, gender equity, and advocacy.

The climate survey not only highlighted persistent challenges in oral health education but also offered each dental school an opportunity to utilize this unique data provided by ADEA to identify impactful solutions for each participating institution, giving them the opportunity to have a collective and positive impact in oral health education. This approach fosters a humanistic environment in oral health education that would benefit stakeholders as we embrace ADEA's commitment to its long history of envisioning a more diverse and impactful oral health workforce.

## DISCLAIMER

This article is based on empirical research. The analysis and interpretations expressed in this piece are solely those of the authors and not intended to represent or reflect the position or viewpoints of the American Dental Education Association, its publisher, or any entities with whom the authors may or may not be affiliated or associated.

[Correction added on 27 May 2025, after first online publication: A disclaimer section has been added to this version.]
